# Development of an Artificial Intelligence-Based System for Evaluating Transthoracic Echocardiographic Imaging in Focus Cardiac Ultrasonography

**DOI:** 10.3390/diagnostics16071032

**Published:** 2026-03-30

**Authors:** Daigo Ikeda, Sanshiro Togo, Shogo Tsuge, Shu Ohya, Yuki Sugiura, Masaya Honda, Taiki Hosokawa, Kenshin Suzuki, Katsumasa Nakamura, Yuki Kurita, Kazuki Tamura, Takeji Saitoh

**Affiliations:** 1 Faculty of Medicine, Hamamatsu University School of Medicine, Hamamatsu 431-3192, Shizuoka, Japan; daigo-ikeda-doctengineer@outlook.jp (D.I.);; 2 Next Generation Creative Education Center for Medicine, Engineering and Informatics (Nx-CEC), Hamamatsu University School of Medicine, Hamamatsu 431-3192, Shizuoka, Japan; 3 Department of Radiation Oncology, Hamamatsu University Hospital, Hamamatsu 431-3192, Shizuoka, Japan; 4 Department of Regenerative and Infectious Pathology, Hamamatsu University School of Medicine, Hamamatsu 431-3192, Shizuoka, Japan; 5 Institute of Photonics Medicine, Hamamatsu University School of Medicine, Hamamatsu 431-3192, Shizuoka, Japan; 6 Graduate School of Engineering Science, Yokohama National University, Yokohama 240-8501, Kanagawa, Japan

**Keywords:** transthoracic echocardiography, artificial intelligence, focus cardiac ultrasound, medical education

## Abstract

**Background/Objectives:** Transthoracic echocardiography (TTE) is a non-invasive tool for real-time assessment of cardiac motion and blood flow. It is widely used in emergency and bedside settings as a Focus Cardiac Ultrasound (FoCUS) device. However, standardized training methods and adequate educational environments are limited. **Methods:** A TTE image assessment artificial intelligence (AI) system was developed in this study, focusing on probe positioning and image quality for non-supervised TTE practice. **Results:** The view classification model achieved a high F1-score of 0.956. The position evaluation model achieved F1-scores of 0.678, 0.864, and 0.831 for the parasternal long-axis, parasternal short-axis, and apical four-chamber views, respectively. The quality evaluation model achieved F1-scores of 0.674, 0.845, and 0.746. Combining the position and quality models improved the F1-score for the parasternal long-axis view to 0.714, showing the benefit of integrating views with lower baseline performance. **Conclusions:** This study presents a novel AI-based educational system that assesses probe position and image quality in TTE. The model was developed using a custom dataset of healthy young adults that reflects beginner-level training scenarios, including many suboptimal images similar to those commonly acquired by novices. The proposed framework, which integrates position and quality models, lays the groundwork for future AI-assisted ultrasound training, particularly in unsupervised or resource-limited settings.

## 1. Introduction

Transthoracic echocardiography (TTE) is an essential diagnostic tool in cardiovascular medicine that enables the real-time assessment of cardiac motion and hemodynamics without causing pain or exposing patients to radiation. When TTE is performed by non-cardiology specialists for rapid diagnosis and initial treatment decision-making, it is termed Focus Cardiac Ultrasound (FoCUS). FoCUS is increasingly recognized as an essential skill for physicians, particularly in the emergency department, the intensive care unit (ICU), and primary care settings [1,2,3,4,5]. Globally, there is a growing emphasis on integrating ultrasound education, including TTE, into undergraduate medical training, with widespread adoption expected among medical students [6].

FoCUS is a brief bedside screening examination mainly conducted by non-cardiology specialists. Its primary goal is to prevent the oversight of common or potentially life-threatening conditions by focusing on a limited number of essential findings using simple and fundamental techniques [7]. Unlike comprehensive echocardiography conducted by ultrasound specialists, FoCUS is not designed to provide a detailed evaluation of the entire heart or quantify parameters, including left ventricular ejection fraction or transvalvular flow velocities.

A significant challenge for beginners in FoCUS training is determining whether the image they obtain represents an appropriate cross-sectional view. Typically, learners acquire this skill through hands-on sessions with ultrasound instructors, who provide direct feedback on image quality. However, tools that are currently available for evaluating image appropriateness are few, making it difficult for beginners to train effectively without the supervision of experienced practitioners. In Japan, many physicians receive FoCUS training during their residency; nevertheless, the limited availability of instructors or technicians in some institutions has led to junior residents completing their programs without acquiring adequate FoCUS skills [7].

Recently, advancements in artificial intelligence (AI) have improved image recognition technology. Notably, research has focused on tasks, including standard view classification, which involves identifying commonly depicted views on TTE, such as the parasternal long-axis view, parasternal short-axis view at the mitral valve level, and apical four-chamber view [8,9,10,11,12,13,14,15]. Additionally, ultrasound devices equipped with AI-guided scanning assistance have become available, helping to acquire appropriate cross-sectional views. However, the underlying algorithms differ among manufacturers and mostly depend on specific ultrasound systems.

There are two essential elements for acquiring optimal TTE images in FoCUS. The first is the position of the probe required to obtain the intended view. The second is whether the anatomical structures necessary for FoCUS were adequately visualized. Some factors, including respiratory motion, imaging artifacts, insufficient gel application, and inappropriate display of structures at their expected locations, can impair the second element. In this study, these two elements are referred to as “position” and “quality,” respectively. Deficiencies in any of these elements can hinder rapid and accurate diagnosis in clinical settings. Skilled sonographers achieve optimal imaging by understanding the target view and continuously adjusting the position and orientation of the probe based on real-time image feedback. Trainees must learn to recognize optimal views and infer the necessary adjustments to the probe based on the images they observe to acquire this level of skill.

In this study, a TTE image assessment AI as an educational tool was developed to help beginners acquire FoCUS skills. A two-step framework was established for this purpose. Step 1 involves classifying the TTE images into three standard views. Step 2 involves evaluating whether each image corresponds to an optimal cross-sectional view and is of sufficient quality. Completing both steps enables the identification of images that are suitable for FoCUS.

Various studies have addressed Step 1 (the prerequisite task), which involves classifying standard echocardiographic views [8,9,10,11,12,13,14,15,16]. From a methodological perspective, deep learning approaches—particularly convolutional neural networks (CNNs) such as ResNet, VGG, and Inception, as well as neural architecture search (NAS)—have become the standard for automated view classification. These models achieve high classification accuracies, often exceeding 95%, and have demonstrated robustness across diverse clinical scenarios, including point-of-care ultrasound (POCUS), contrast-enhanced echocardiography, and handling cardiac motion via spatial-temporal or graph-based constraints.

Despite these strengths, a key limitation of existing tools is their reliance on datasets composed primarily of clinically optimal, properly aligned standard views. Their primary objective is to identify which standard view is depicted, rather than to detect how the probe is misaligned. Consequently, they cannot evaluate the typical suboptimal images produced by novice trainees during hands-on practice. Some studies have also investigated the use of neural networks to evaluate the image quality in TTE as part of Step 2 (the primary task) [16,17,18]. However, estimating the position of the probe from an image requires a unique dataset that includes intentionally misaligned or suboptimal images. Because existing automated tools were not designed or trained to output fine-grained probe deviation classes (e.g., distinguishing between specific sliding, tilting, or rotating errors from a reference view), a direct empirical comparison between our implementation and other existing tools using the same data is not feasible; the classification tasks and output labels are fundamentally different.

Three types of image-classification tasks were conducted in this study. The first image classification task focused on achieving the previously described Step 1, where a view classifier was used to identify the three cross sections: parasternal long-axis view, parasternal short-axis view at the mitral valve level, and apical four-chamber view. The second task involved estimating positional deviations from the correct probe position using echocardiographic images. This position classification task used a position classifier to categorize 15 types of deviations in the parasternal long-axis view, 19 in the parasternal short-axis view, and 18 in the apical four-chamber view. The third task focused on evaluating the visual quality of the displayed images by categorizing them into four levels–best, acceptable, poor, and bad–using a quality classifier. The position and quality classifiers were designed to achieve Step 2 and were individually trained based on the view classifier. Subsequently, the results predicted by the position and quality classifiers were combined or threshold-processed to enhance the performance of the position classification using quality classification as a weighting factor. The classification results were evaluated using Venn diagrams under two conditions: one where all images, regardless of quality, were defined as targeted images and the other where only high-quality images (best or acceptable) were defined as targeted images. The latter metric was intended to identify images suitable for FoCUS. Finally, Step 3 is discussed based on the results of the position classifier for non-optimal cross sections.

We engaged in an engineering optimization task aimed at the practical implementation of an AI system that assesses TTE image quality based on the FoCUS criteria without requiring supervision from expert sonographers or technicians. While we utilized an existing AI architecture for system development, the novelty of this study lies in the construction of a framework that integrates a unique dataset with distinct evaluation models. In particular, images where the probe position deviated from the optimal cross-sectional plane were collected. Classifiers based on these images were constructed to estimate the probe position and assess diagnostic image quality.

## 2. Methods

The Ethics Committee of Hamamatsu University School of Medicine (EC HUSM) and the Conflict-of-Interest Management Committee (Approval No. 22-121) approved this study. All methods were carried out in accordance with relevant institutional guidelines and regulations. The study was specifically designed and conducted in accordance with the Declaration of Helsinki. All the participants provided written informed consent. The authors declare that they have no conflicts of interest associated with this study.

### 2.1. Collection and Annotation of Training and Validation Datasets

In this study, two datasets were obtained: one for training the view classification model, and the other was used as the main dataset to develop the position and quality evaluation models. The models developed in this study were designed to assess whether echocardiographic images acquired during practice sessions by beginner-level FoCUS trainees are appropriate views. Data were collected exclusively from healthy young adults, representing the target population of beginner users of FoCUS. A scanning protocol was developed to systematically acquire various intentionally suboptimal images.

#### 2.1.1. Collection of the View Classification Model Dataset

Between June and August 2023, data were collected from six medical students proficient in TTE, under the supervision of a cardiologist certified by the Japanese Circulation Society. A method was adopted to intentionally capture images that deviated from optimal views, starting from reference standard views.

**Figure 1:**
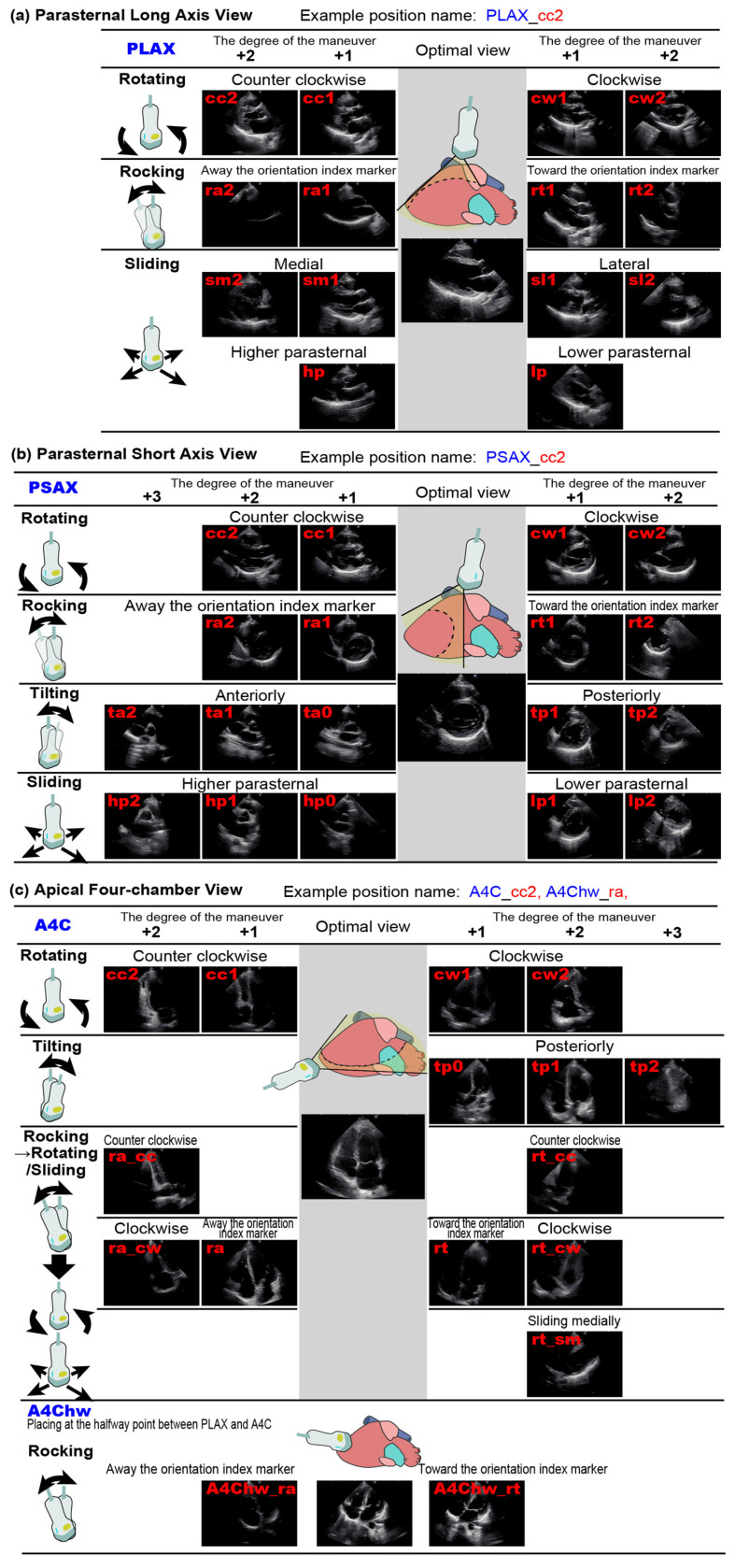
Position Labels for Images Captured in This Study. The optimal cross-sectional view for each position was defined as the starting point, and suboptimal views were obtained by applying rotating, rocking, tilting, or sliding maneuvers to the transducer. Non-optimal views were labeled by appending an underscore to the name of the optimal view, followed by an abbreviation of the maneuver (“cc” for counterclockwise rotation) and the degree of the maneuver (0, 1, or 2). (**a**) Parasternal long-axis view. (**b**) Parasternal short-axis view at the mitral valve level. (**c**) Apical four-chamber view.

**Figure 2:**
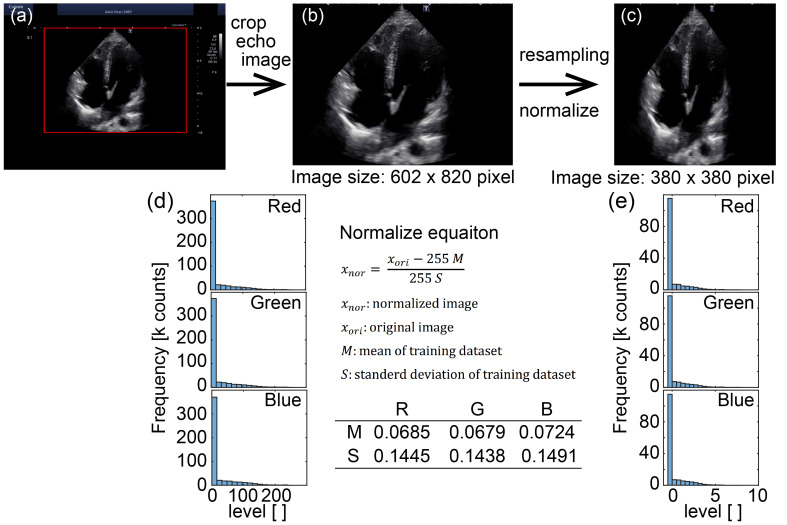
Overview of Preprocessing and Normalization for Images. Preprocessing pipeline for echocardiography images used as inputs. (**a**) Screenshot of the diagnostic ultrasound system. The region of interest (ROI) indicated by the red rectangle is extracted. (**b**) Cropped grayscale echo image from the ROI (image size: 602 × 820 pixels). (**c**) Final CNN input image after resampling to a square format and intensity normalization (image size: 380 × 380 pixels). (**d**,**e**) Pixel-intensity histograms of the cropped image in (**b**) and (**c**), respectively.

**Figure 3:**
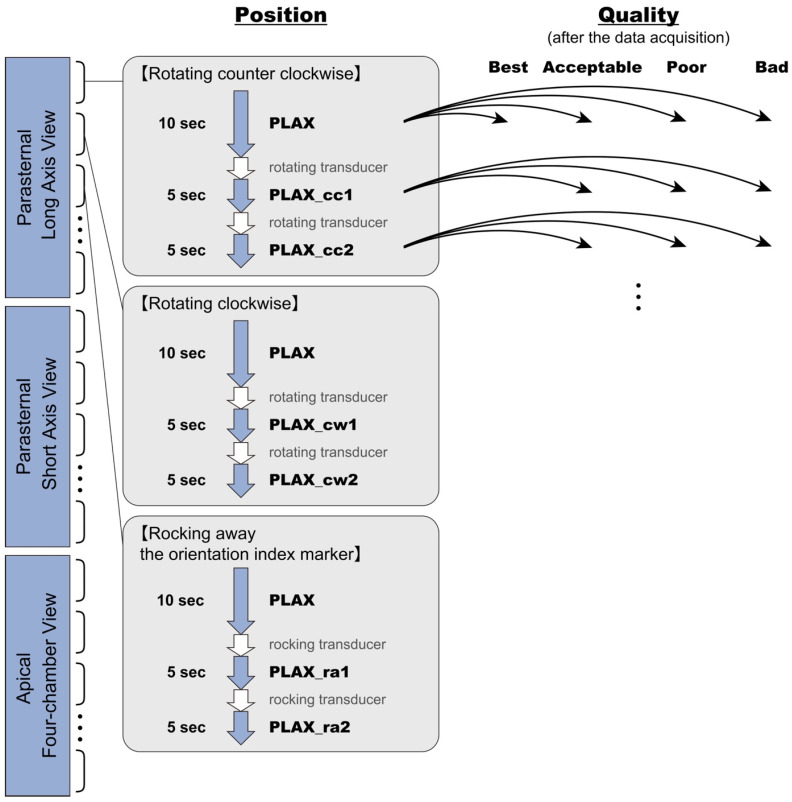
Overview of Data Acquisition and Labeling Methods. The ultrasound data collection was divided into three parts: the parasternal long-axis view, the parasternal short-axis view at the mitral valve level, and the apical four-chamber view. For each standard view, a series of scans was conducted, consisting of seven to eight sequences per part. Each sequence began by continuously capturing the optimal view for 10 s (or 5 s), followed by transducer maneuvers to acquire deviated images. For instance, in the counterclockwise rotating sequence for the parasternal long-axis view part, the PLAX view was recorded for 10 s, followed by a counterclockwise rotation to capture the PLAX_cc1 view for 5 s, and further rotation to capture the PLAX_cc2 view for another 5 s. Similarly, clockwise rotations, rocking toward the transducer marker, and other maneuvers were included in the sequences. The arrows indicate that each image has been assigned a quality label.

The dataset included three standard views: the parasternal long-axis (PLAX) view, parasternal short-axis (PSAX) view at the mitral valve level, and apical four-chamber (A4C) view. Each view was categorized into seven to eight scanning sequences. Each sequence began with the optimal view displayed for 5–10 s, followed by systematic probe adjustments to acquire intentionally deviated images (Figure 1, Figure 2 and Figure 3; Supplementary Table S1). For instance, in the counterclockwise rotation sequence for the PLAX view, the optimal view was shown for 10 s, followed by PLAX_cc1 and PLAX_cc2, each recorded for 5 s. This process was used across all three standard views. Scanning techniques, including rotating, sliding, tilting, and rocking, were not defined by the physical angle of the probe but by the visualization of anatomical structures on the ultrasound images, according to the criteria shown in Supplementary Table S1. In the PLAX view, the images obtained through “sliding lower parasternal” and “tilting posteriorly” were found to be similar in appearance, making it difficult to distinguish them. Therefore, only the “sliding lower parasternal” maneuver was used during data acquisition. Similarly, when multiple scanning methods produced nearly indistinguishable images, only one image was selected for data collection. Consequently, the specific scanning protocols varied slightly across the PLAX, PSAX, and A4C views.

The data acquisition process was conducted using the Aplio verifia™ (Canon Medical Systems, Ohtawara, Japan) with a PST-28BT probe, recording videos at 60 fps. Before data collection, the imaging personnel underwent training in probe manipulation and followed predefined procedures.

The recorded video data were subsequently annotated by four medical students, with the supervision of a cardiologist. Each video was labeled and cross-checked by three separate annotators to ensure the quality of the annotation. View labels were assigned according to the section during data collection, whereas position labels were determined by cross-referencing video timestamps with probe manipulations within each series. Segments with unintended probe displacement or significant image distortion were excluded from the dataset.

#### 2.1.2. Collection of the Main Dataset

Between January and May 2023, the main dataset for the position and quality evaluation models was collected using the same methods and equipment as the pretrained model dataset (Figure 1, Figure 2 and Figure 3). Data acquisition was conducted using the Aplio verifia™ (Canon Medical Systems) with a PST-28BT probe. The annotation process was conducted by the four medical students who worked on the view classification model dataset.

In addition to the position labels, the main dataset included quality labels categorized into four levels: best, acceptable, poor, and bad. The criteria for quality evaluation were according to the American Society of Echocardiography guidelines [19], with acceptable or higher quality defined as acceptable (Supplementary Table S2). Best: Anatomical structure required for the FoCUS was clearly visible during systole and diastole. Acceptable: At least one systole or diastole showed a partial depiction of the required anatomical structures. Poor or Bad: Systole and diastole show incomplete or missing depictions of the required anatomical structures.

The main dataset was categorized into two parts: 15 participants were set aside as the test dataset, and the remaining participants were used for training and validation.

### 2.2. Dataset Processing

**Figure 4:**
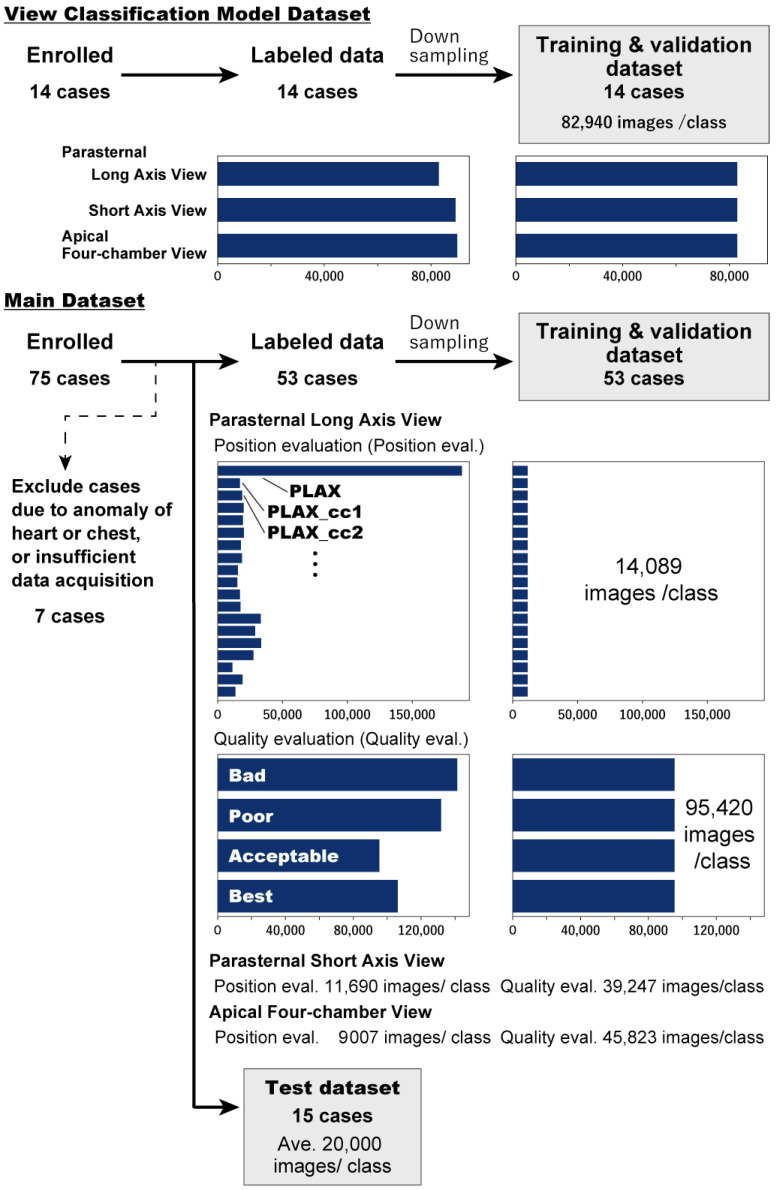
Data Processing Workflow. This figure shows the process of generating training and testing datasets from the collected video data. The arrows indicate the flow of data selected from the enrolled participants.

For training and inference, standardized preprocessing procedure to the echocardiography frames (Figure 2) was applied. First, we extracted the ROI from the ultrasound system screen by cropping the area indicated by the red rectangle (Figure 2a), resulting in a rectangular image (602 × 820 pixels; Figure 2b). The cropped image data were then randomly undersampled to ensure an equal number of samples per class (Figure 4, Supplementary Table S4).

### 2.3. Learning Method

#### 2.3.1. Computational Environment

All experiments were conducted using Python version 3.8.10. The following libraries were used: torch v1.13.0, torchvision v0.14.0, pillow v9.3.0, scikit-learn v1.1.3, scikit-image v0.19.3, pandas v1.5.2, numpy v1.23.5, seaborn v0.12.1, cuda v11.7.99, cudnn v8.5.0.96, opencv-python v4.6.0.66, accelerated v0.22.0, timm v0.6.12, and albumentation v1.3.0. The experiments were conducted on two RTX A6000 GPUs (NVIDIA, Santa Clara, CA, USA), each with 48 GB of memory, to ensure sufficient computational resources.

#### 2.3.2. Training View Classification Model

As the first step in this study, a model was developed to classify echocardiographic images into three standard views: the PLAX view, PSAX view at the mitral valve level, and A4C view (the view classification model). The position and quality evaluation models were built using the view classification model as a pretrained backbone.

The view classification model was used as a pretrained model for the position and quality evaluation models for two reasons. First, pretraining on a relatively simple task facilitates adaptation to subsequent tasks. Second, constructing position and quality evaluation models directly from existing pretrained models, such as ImageNet, requires substantial computational resources. The view classification model avoids this issue by improving computational efficiency. Existing image recognition models, such as ImageNet, can be used; however, the domain gap between natural and echocardiographic images poses challenges in achieving high classification accuracy. Additionally, some studies have leveraged open datasets of echocardiographic images; nevertheless [20], there are legal constraints pertaining to their use, limiting their versatility. Therefore, in this study, the view classification model was used as a pretrained model for the position and quality evaluation models.

To obtain a fixed input size compatible with the network, the cropped image was resampled to a square image (380 × 380 pixels; Figure 2c). We then normalized the pixel intensities using the training-dataset statistics to reduce inter-scan intensity variability and stabilize optimization. Mean and standard deviation values were computed from the training dataset. The resulting histograms (Figure 2d,e) show that this procedure shifts and scales the pixel-value distribution toward a near-zero mean with reduced spread, producing standardized inputs for the network.

The following augmentations were used: random rotation within ±20° (Rotate) and random cropping within 90–100% of the original size while maintaining an aspect ratio of 1.36 (RandomResizedCrop). These augmentations were applied to 30% of images in each batch. Following preprocessing, mean values and standard deviations were used to normalize the images.

**Figure 5:**
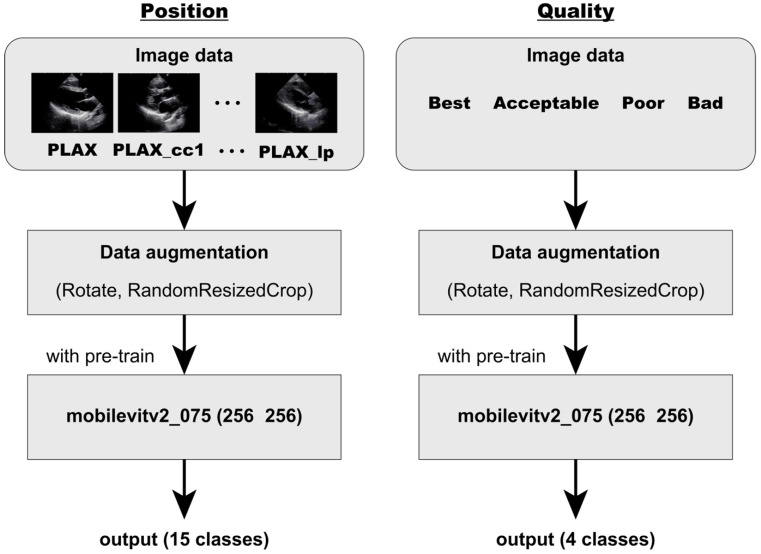
Training Methods for Position and Quality Evaluation Models. The figure shows the training process for the position evaluation model (parasternal long-axis view) and the quality evaluation model. Training images were subjected to data augmentation with a 30% probability and then input into the mobileViTv2_075 model. The model outputs the class probabilities for each evaluation.

The images were subsequently input into MobileViTv2_075 [21,22] (Figure 5). The backbone architecture was selected based on a comprehensive comparative evaluation of several lightweight models, including MobileViTv2_075, conducted under identical training and validation conditions. In addition to primary task performance, inference latency, model size, and the propensity for overfitting were systematically assessed (Supplementary Table S3). Vision Transformer (ViT) is a deep learning architecture mainly used for image classification. Unlike convolutional neural networks (CNNs), which perform convolution operations on small local regions of an image (3 × 3 or 5 × 5 pixels) and are, therefore, limited to learning local features, ViTs uses a self-attention mechanism that enables them to process the entire image simultaneously. This enables the model to associate local features directly with the global image context, allowing learning that considers the entire image structure.

ViTs generally require more computational resources than CNNs; nonetheless, MobileViT was developed as a lightweight alternative that maintains similar accuracy with a reduced computational cost. In this study, MobileViTv2_075, an even more efficient version, was used to facilitate real-time image evaluation using smartphones and tablets.

The dataset was divided into training and validation sets using a stratified grouped hold-out approach, with subject identifiers treated as grouping variables. Model training was conducted to ensure independence at the subject level. The split ratio and random seed are specified in the main text. The optimization yielded an ideal batch size of 256 and a learning rate of 0.001. The view classification model was trained for 150 epochs, the position evaluation models for 200 epochs, and the quality evaluation models for 50 epochs. The model corresponding to the epoch with the highest F1-score was retained. The remaining hyperparameters are shown in Table 1 and Table 2.

#### 2.3.3. Training Position and Quality Evaluation Models

The position and quality evaluation models for each standard view were trained and validated using a view-classification model. Only data corresponding to each specific view were used for training; for instance, only images from the PLAX view were used for the position evaluation model.

Both models were built using mobileViTv2_075 (input size: 256 × 256 pixels) as the backbone (Figure 5). Identical hyperparameters were used for both the position evaluation model and the quality evaluation model (Table 2). The model corresponding to the epoch with the highest F1-score was retained.

### 2.4. Evaluation Methods

#### 2.4.1. Evaluation Metrics

The primary evaluation metrics included a recall, a precision, and an F1-score, which showed the harmonic mean of precision and recall for the position evaluation model in determining the correct anatomical planes for each view. Recall, precision, and F1-score of the quality evaluation model for identifying images of acceptable quality (best or acceptable) were measured.

Secondary metrics included a recall, a precision, and an F1-score for the pretrained classification model. Recall, precision, and F1-score for position prediction when combining the position and quality evaluation models were measured. Macro-average recall, precision, and F1-score for identifying images that met positional and quality criteria across all views were calculated.

#### 2.4.2. Test Case Evaluation

The performances of the view classification, position evaluation, and quality evaluation models were assessed using the test data from 15 participants excluded from the training set. The macro-averaged recall, precision, and F1-score were computed for each model.

#### 2.4.3. Combining Position and Quality Evaluation Models

The potential of combining the position and quality evaluation models to improve positional predictions was investigated. The intersections and unions of the positional and quality evaluation results (best or acceptable) were used, while the corresponding recall, precision, and F1-scores were calculated.

#### 2.4.4. Identifying FoCUS-Usable Images

A model for identifying FoCUS-usable images (those meeting the positional and quality criteria) was evaluated. The accuracy of the model was assessed using recall, precision, and F1-scores according to the intersection and union of the position and quality evaluation models.

## 3. Results

### 3.1. Participants Characteristics

Table 3 shows the characteristics of the participants whose data were used to train the position, quality, and view classification models. Overall, 281,534 frames from 14 participants (maximum: 22,202 frames; minimum: 1918 frames) were used exclusively for training. An additional 425,781 frames from 15 participants (an average of 28,385 frames per subject) were used for testing.

Among the 75 participants initially enrolled in the position and quality evaluation models, seven were excluded owing to factors such as pectus excavatum or insufficient image acquisition. The remaining 1,886,225 frames from 68 participants were grouped as follows: 595,571 frames from 53 participants (average: 11,237 frames per participants after undersampling) were used for training and validation, and 425,781 frames from 15 participants (average: 28,385 frames per subject) were used for testing.

The undersampled data used for training are as follows (Figure 4): view classification model, 82,940 frames per class; position evaluation model, parasternal long-axis, 14,089 frames per class; parasternal short-axis, 11,690 frames per class; apical four-chamber, 9007 frames per class; quality evaluation model, parasternal long-axis, 95,420 frames per class; parasternal short-axis, 39,247 frames per class; and apical four-chamber, 45,823 frames per class.

For testing, the view classification model used 425,781 frames (average: 30,413 frames per participants), while the Position and Quality evaluation models used 141,927 frames (average: 9461 frames per participants). The distribution of quality labels according to their position in the test data is shown in Table 4.

### 3.2. Results of the View Classification Model

**Figure 6:**
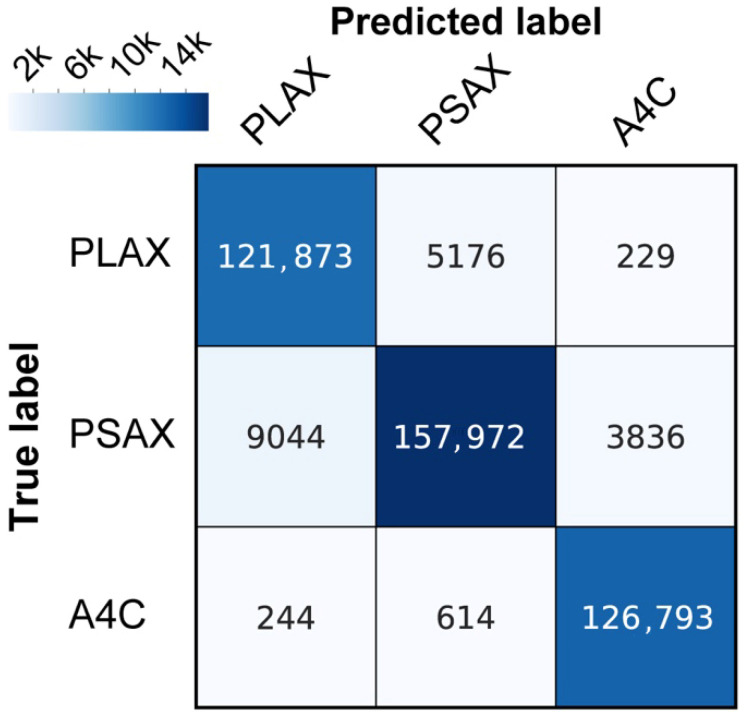
Results of the View Classification Model. The recall, precision, and F1-score of the view classification model for classifying three standard views were 0.959, 0.954, and 0.956, respectively. Among the 425,781 images from the 15 test participants in the dataset, 19,143 images (4.5%) were misclassified as incorrect views.

Figure 6 shows a confusion matrix for classifying echocardiographic images into three standard views: the parasternal long-axis, parasternal short-axis, and apical four-chamber views. Numbers within each square indicate the number of images in each category.

Overall, 121,873 images with the true label “PLAX” were correctly classified as “PLAX.” However, 5176 images with the true label “PLAX” were misclassified as “PSAX”, and 229 images with the true label “PLAX” were misclassified as “A4C”. Similarly, 9044 images with the true label “PSAX” were misclassified as “PLAX,” while 157,972 images with the true label “PSAX” were correctly classified. Additionally, 3836 images with the true label “PSAX” were misclassified as “A4C.” For the true label “A4C,” 244 images were misclassified as “PLAX,” and 614 images with the true label “A4C” were misclassified as “PSAX,” while 126,793 images with the true label “A4C” were correctly classified. The rows indicate true labels, and the columns indicate predicted labels. Color intensity corresponds to the number of images, as indicated by the color bars.

Evaluating the view classification model designed to categorize the three standard views achieved an accuracy of 0.955, a recall of 0.959, a precision of 0.954, and an F1-score of 0.956 (Figure 6). Among the 425,781 images in the test dataset (15 participants), 19,143 images (4.5%) were misclassified as incorrect. Given the high F1-score, the view classification model was considered suitable as a pretrained model and was subsequently used to develop the position and quality evaluation models.

### 3.3. Results of the Position Evaluation Model

**Figure 7:**
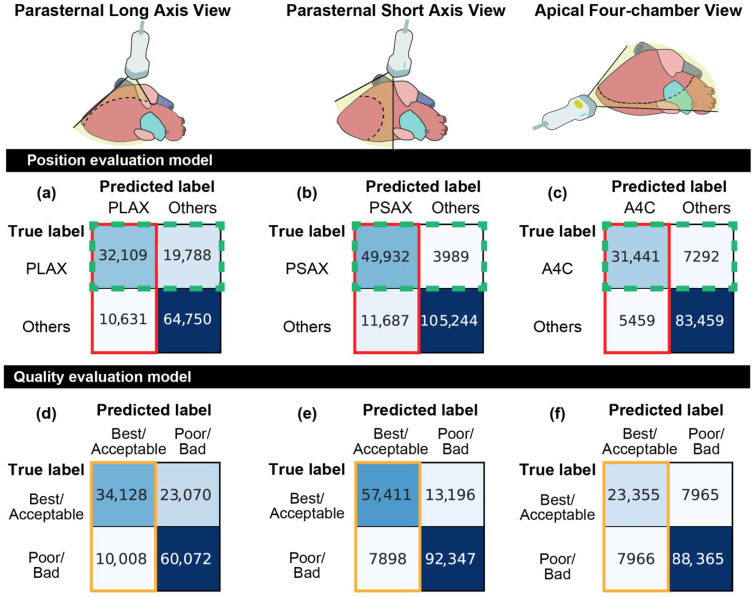
Inference Results of the Position Evaluation Model: The vertical axis represents the actual labels, and the horizontal axis represents the predicted labels. Each cell color indicates the number of samples. (**a**) Position evaluation model for parasternal long-axis view. (**b**) Position evaluation model for parasternal short-axis view. (**c**) Position evaluation model for apical four-chamber view. Inference Results of the Quality Evaluation Model: The vertical axis represents the actual labels, and the horizontal axis represents the predicted labels. Each cell color indicates the number of samples. (**d**) Quality evaluation model for parasternal long-axis view. (**e**) Quality evaluation model for parasternal short-axis view. (**f**) Quality evaluation model for apical four-chamber view. Three colored outlines (green, red, and orange) enclose parts of the confusion matrix and indicate the combinations corresponding to the regions in the Venn diagram in Figure 8.

The performance of the position evaluation models, which assess whether the probe is correctly positioned to capture optimal views, including the PLAX view, PSAX view, and A4C view, was evaluated using the test dataset (Table 5, Figure 7).

**Figure 8:**
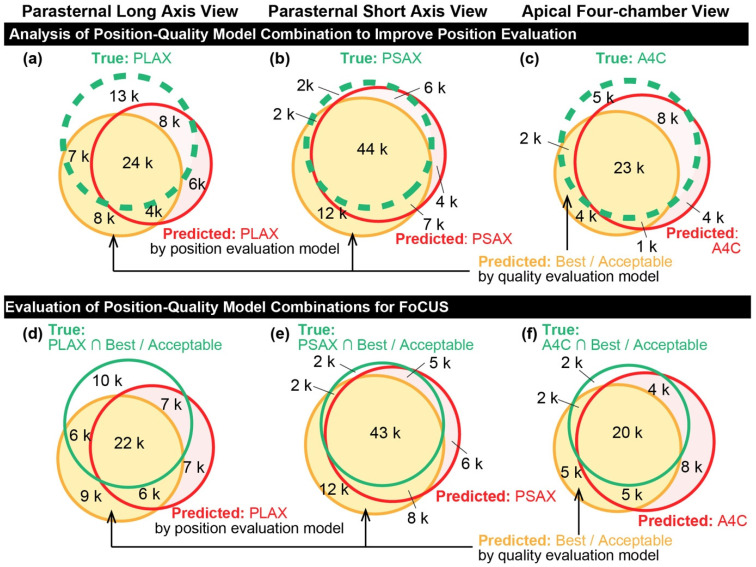
Venn Diagrams of the Predicted Appropriate Sections by Position and Quality Evaluation Models Compared with the Ground Truth: The green dashed line, red solid line, and purple solid line represent the regions of the ground truth labels for appropriate sections, the predicted regions by the position evaluation model, and the predicted regions by the quality evaluation model, respectively. (**a**) Parasternal long-axis view. (**b**) Parasternal short-axis view. (**c**) Apical four-chamber view. Venn Diagrams of the Predicted Appropriate Sections with Best or Acceptable Quality by Position and Quality Evaluation Models Compared with the Ground Truth: The ochre dashed line, red solid line, and purple solid line represent the regions of the ground truth labels for appropriate sections with acceptable quality (best or acceptable), the predicted regions by the position evaluation model, and the predicted regions by the quality evaluation model, respectively. (**d**) Parasternal long-axis view. (**e**) Parasternal short-axis view. (**f**) Apical four-chamber view.

Figure 7 shows the confusion matrices for the position evaluation model applied separately to each standard view: (a) PLAX, (b) PSAX, and (c) A4C views. The matrix compares the number of images correctly classified as “optimal view” versus those classified as “other” positions for each view.

For the PLAX model, 32,109 images with the true label “PLAX” were correctly identified as optimal, while 19,788 were misclassified as “others.” Conversely, 10,631 images from other positions were misclassified as “PLAX,” and 64,750 were correctly classified as “others.” The model achieved an accuracy of 0.761, a recall of 0.619, a precision of 0.751, and an F1-score of 0.679 when evaluating the proper sections. When including optimal and non-optimal sections (full range of positional classes), the model achieved a recall of 0.492, a precision of 0.477, and an F1-score of 0.474 (see Supplementary Figure S1(I) online).

For the PSAX model, 49,932 images with the true label “PSAX” were correctly identified, while 3989 were misclassified as “others.” Among the images from other positions, 11,687 were incorrectly classified as “PSAX” and 105,244 were correctly identified as “others.” The accuracy, recall, precision, and F1-score for the proper sections were 0.908, 0.926, 0.810, and 0.864, respectively. When including optimal and non-optimal sections, the recall, precision, and F1-score were 0.656, 0.680, and 0.660, respectively (see Supplementary Figure S1(II)).

For the A4C model, 31,441 images with the true label “A4C” were correctly classified, while 7292 were misclassified as “others.” Conversely, 5459 images from other positions were incorrectly classified as “A4C,” and 83,459 were correctly classified as “others.” The model achieved an accuracy of 0.900, a recall of 0.812, a precision of 0.852, and an F1-score of 0.831 for the proper sections. When including both optimal and non-optimal sections, the recall, precision, and F1-score were 0.585, 0.614, and 0.573, respectively (see Supplementary Figure S1(III)).

In all matrices, the rows correspond to the true labels and the columns to the predicted labels. Color intensity represents the number of images, as shown by the color scale above each matrix.

### 3.4. Results of the Quality Evaluation Model

The performance of the quality evaluation model, which assessed whether key anatomical structures are visible without prominent artifacts, was tested using four quality levels (best, acceptable, poor, and bad) on the test dataset (Table 6, Figure 7).

Figure 7 shows the confusion matrices for the quality evaluation model applied separately to the (d) PLAX, (e) PSAX, and (f) A4C views. In each matrix, true labels are categorized as “Best/Acceptable” or “Poor/Bad,” with predictions shown in the same categories.

For the PLAX view, 34,128 images with the true label “Best/Acceptable” were correctly classified, while 23,070 were misclassified as “Poor/Bad.” Among the images labeled “Poor/Bad,” 10,008 were misclassified as “Best/Acceptable,” while 60,072 were correctly classified. The model achieved an accuracy of 0.740, a recall of 0.597, a precision of 0.773, and an F1-score of 0.674 when assessing combined “best” and “acceptable” quality.

For the PSAX view, 57,411 images with the true label “Best/Acceptable” were correctly identified, while 13,196 were misclassified as “Poor/Bad.” Among the images labeled “Poor/Bad,” 7898 were incorrectly classified as “Best/Acceptable,” and 92,347 were correctly identified. The corresponding scores were an accuracy of 0.877, a recall of 0.813, a precision of 0.879, and an F1-score of 0.845.

For the A4C view, 23,355 images with the true label “Best/Acceptable” were correctly classified, while 7965 were misclassified as “Poor/Bad.” Among the “Poor/Bad” images, 7966 were incorrectly labeled as “Best/Acceptable,” and 88,365 were correctly classified. The corresponding scores were an accuracy of 0.875, while recall, precision, and F1-score were 0.746 across all metrics.

The rows indicate true labels, whereas the columns indicate predicted labels. Color intensity corresponds to the number of images, as shown by the scale bar above each matrix.

### 3.5. Analysis of Position-Quality Model Combination to Improve Position Evaluation

The combination of the position and quality evaluation models was compared with the standalone position evaluation model (Table 7, Figure 8a–c). The Venn diagrams show the overlap between the ground truth labels (green dashed circles), regions predicted by the position evaluation model (red solid circles), and regions predicted by the quality evaluation model (purple solid circles) for each standard view: (a) PLAX, (b) PSAX, and (c) A4C. The corresponding areas in the confusion matrices in Figure 7 are aligned with the Venn diagrams in Figure 8a–c. The two confusion matrices at the bottom of the figure represent the results of the position evaluation model (left) and quality evaluation model (right).

For the PLAX view, 51,897 images were labeled using PLAX. The position evaluation model predicted 42,740 images as PLAX, while the quality evaluation model predicted 44,136 images as “Best/Acceptable.” The intersection of the two models produced 28,722 images, of which 24,419 were correctly labeled as PLAX. Using this intersection, the recall, precision, and F1-score were 0.471, 0.850, and 0.606, respectively. Using the union, 58,154 images were produced, and the corresponding metrics were 0.757, 0.676, and 0.714, representing an improvement in the F1-score compared to the position model alone (0.679).

For the PSAX view, 53,921 images were labeled with PSAX. The position evaluation model predicted 61,619 as PSAX, while the quality evaluation model predicted 65,309 as “Best/Acceptable.” The intersection yielded 51,265 images with a recall, a precision, and an F1-score of 0.815, 0.857, and 0.835, respectively. The union yielded 75,663 images with a recall, precision, and F1-score of 0.964, 0.687, and 0.802, respectively, both of which were lower than the 0.864 achieved by the position model alone.

For the A4C view, 38,733 images were labeled as A4C. The position evaluation model predicted 36,900 as A4C, while the quality evaluation model predicted 31,321 as “Best/Acceptable.” This intersection yielded 24,667 images with a recall, a precision, and an F1-score of 0.602, 0.946, and 0.736, respectively. The union yielded 43,554 images with a recall, a precision, and an F1-score of 0.869, 0.773, and 0.818, respectively. However, both approaches showed lower F1-scores than that of the positional model alone (0.831).

Table 7 shows the estimation results. Collectively, these findings indicate that combining the position and quality evaluation models enhanced the F1-score for the PLAX view, especially when using the union approach, while for the PSAX and A4C views, the position model alone outperformed the combined approach.

### 3.6. Evaluation of Position-Quality Model Combinations for FoCUS

To identify images suitable for FoCUS, position and quality evaluation models were combined to classify images meeting the appropriate position and quality criteria (labeled as either “best” or “acceptable”). The inference results are shown in Table 8 and Figure 8d–f.

Figure 8d–f shows the results of combining the position and quality evaluation models to identify the images suitable for FoCUS. The green dashed circles represent the ground truth labels of images classified as appropriate position and “Best/Acceptable” quality, the red solid circles indicate the regions predicted by the position evaluation model, and the purple solid circles indicate the regions predicted by the quality evaluation model.

For the PLAX view, 45,235 images were labeled as PLAX with Best/Acceptable quality. The position evaluation model predicted 42,740 images as PLAX, whereas the quality evaluation model predicted 44,136 images as best or acceptable. The intersection of the two models produced 28,722 images, of which 22,308 were correctly labeled as PLAX with the Best/Acceptable quality. The union resulted in 58,154 images, of which 35,647 were correctly labeled as PLAX with the Best/Acceptable quality. The calculated metrics were as follows: recall 0.493, precision 0.777, and F1-score 0.603 for the intersection, and recall 0.788, precision 0.613, and F1-score 0.690 for the union.

For the PSAX view, 51,222 images were labeled as PSAX with Best/Acceptable quality. The position evaluation model predicted 61,619 images as PSAX, whereas the quality evaluation model predicted 65,309 images as Best/Acceptable. The intersection of the two models produced 51,265 images, of which 43,199 were correctly labeled as PSAX with the Best/Acceptable quality. The union resulted in 75,663 images, of which 49,714 were correctly labeled as PSAX with the Best/Acceptable quality. The calculated metrics are as follows: recall = 0.843, precision = 0.843, and F1-score 0.843 for the intersection, and recall 0.971, precision 0.657, and F1-score 0.784 for the union.

For the A4C view, 28,039 images were labeled as A4C with Best/Acceptable quality. The position evaluation model predicted 36,900 images as A4C, whereas the quality evaluation model predicted 31,321 images as Best/Acceptable. The intersection of the two models yielded 24,667 images, of which 20,134 were correctly labeled as A4C with Best/Acceptable quality. The union resulted in 43,554 images, of which 26,278 were correctly labeled as A4C with Best/Acceptable quality. The calculated metrics were recall 0.718, precision 0.816, and F1-score 0.764 for the intersection, and recall 0.937, precision 0.603, and F1-score 0.734 for the union.

## 4. Discussion

Securing adequate practice time is essential to improve TTE skills. However, owing to the limited availability of clinical instructors, the demand for educational applications that support independent learning is increasing. The core component of these applications is an AI system capable of evaluating the quality of echocardiographic images and the positioning of the ultrasound probe. In this study, we developed and evaluated an image-assessment AI system using a dataset collected prospectively from healthy volunteers. A two-step framework was used to evaluate the performance of the AI system.

In the first step, a view classification model was developed to classify the images into three standard views: PLAX, PSAX, and A4C. The model achieved a high F1-score of 0.956. In the second step, the system evaluated whether the images depicted optimal cross sections of these three standard views and assessed their quality. The F1 scores of the position evaluation models for each standard view were 0.679 for PLAX, 0.864 for PSAX, and 0.831 for A4C. Conversely, the F1-scores of the quality evaluation model were 0.674 for PLAX, 0.845 for PSAX, and 0.746 for A4C. Inference methods that integrate a position-evaluation model with a quality-evaluation model were explored in this study. Among the three standard echocardiographic views, the union of the position and quality evaluation models yielded a higher F1-score in the PLAX view (0.714) than the position model alone (0.679). Conversely, in the PSAX and A4C views, the F1-score achieved by the position evaluation model alone outperformed the union and intersection combinations of the two models. Notably, the F1-score for the PLAX view using the position evaluation model alone was 0.679, over 0.1 points lower than the scores for the PSAX and A4C views, which were 0.864 and 0.831, respectively. These results indicate that combining the position and quality evaluation models improved the F1-score of the PLAX view, thereby compensating for its relatively lower baseline performance.

The position and quality evaluation models were integrated in this study to evaluate FoCUS usable imaging. For the PLAX view, combining the position and quality evaluation models using a union approach enhanced the F1-score to 0.714. For the PSAX view, the intersection approach produced an F1-score of 0.835, while for the A4C view, the union approach achieved an F1-score of 0.818. The proposed position and quality evaluation models and their integration showed adequate accuracy in assessing the appropriate sections.

The aim of this study was to estimate the probe position from echocardiographic images; however, the accuracy of classifying non-optimal cross-sectional views remained relatively low. One contributing factor was the use of multiclass classification within each standard view, involving 15 to 19 different classes. Grouping similar classes based on the degree of deviation, for instance, by combining PLAX_cc1 and PLAX_cc2, may improve the classification performance for non-optimal sections.

Additionally, the quality evaluation was based on the depiction of anatomical structures, some of which were very small in the images. This unique characteristic of TTE images, where small anatomical structures significantly influence quality ratings, poses challenges for developing quality evaluation models. The limited training data compared to other studies also restricted the performance. Data augmentation was used to enhance diversity; nonetheless, its impact was insufficient.

The proposed models were designed for use on tablets and smartphones using MobileViTv2_075 for efficient operation in resource-constrained environments. Recent advancements in image recognition, including Vision–Language Models (VLMs) [23,24], may further improve classification accuracy. Among these, Bootstrapping Language–Image Pre-training (BLIP) is a vision–language pre-training framework that supports both understanding and generation tasks [25]. BLIP enhances supervision by generating synthetic captions and filtering out low-quality samples, thereby improving model robustness. Future work may explore the application of VLMs such as BLIP to further improve image assessment performance.

This study makes three major contributions to the literature. First, it is novel because a unique dataset collected from healthy young adults, reflecting practice scenarios among beginner-level trainees, was used for the development of the AI model. Second, the model was trained using a dataset that included a large number of suboptimal images that deviated from optimal cross-sectional views, similar to those beginners often encounter during image acquisition. This deliberate inclusion of non-ideal images has enabled the development of position classifiers capable of evaluating subtle deviations from optimal views, an aspect often overlooked in previous studies. Furthermore, a quality evaluation was conducted based on anatomical visibility to address the unique imaging characteristics of TTE. Third, our proposed framework that integrates position and quality models improved overall performance.

The performances of the models were rigorously evaluated. Position classifiers achieved strong performance for the PSAX and A4C views; however, combining them with quality classifiers further improved the results for views with lower baseline performance, including the PLAX view. The integration of the position and quality models enabled the identification of images suitable for FoCUS, even without the supervision of an expert. These findings highlight the potential of AI-based systems to support independent simulation-based ultrasound training, particularly in environments with limited access to instructors.

## Figures and Tables

**Table 1:** Overview of Training Methods for Models Developed in This Study.

Model	Optimal View	Total Epoch
Pre-trained	PLAX, PSAX, A4C	150
Position evaluation	PLAX	200
	PSAX	200
	A4C	200
Quality evaluation	PLAX	50
	PSAX	50
	A4C	50

Other training hyperparameters are summarized in Table 2.

**Table 2:** Hyperparameters Used for Model Training in Position Evaluation Model and Quality Evaluation Model.

Parameter	Value
Batch size	256
Learning rate	0.001
Label smoothing	0.2
Warmup steps	5
Weight decay	0.01

**Table 3:** The Characteristics of the Subjects Used in This tudy.

		Training Dataset for View Classification Model	Training Dataset for Position and Quality Evaluation Model	Test Dataset
Number		14	53	15
Age (years) •		22 ± 2.8	23 ± 3.6	22 ± 2.8
Sex				
	Male	13 (92.9)	46 (86.8)	13 (86.7)
	Female	1 (7.1)	7 (13.2)	2 (13.3)
BMI, kg/m^2^ •		22 ± 2.6	21 ± 2.5	22 ± 2.3
Total images		281,534	595,571	425,781

Unless otherwise noted, data are numbers of subjects, with percentages in parentheses. • Data are mean ± standard deviation.

**Table 4:** Distribution of Positions and Quality.

**(I) Parasternal Long-Axis View**						
			**Quality**			
**Position**		**Total**	**Best**	**Acceptable**	**Poor**	**Bad**
Optimal view	PLAX	51,897 (185,140)	28,580 (104,636)	16,655 (52,298)	5431 (25,971)	1231 (2235)
Rotating	PLAX_cc2	9964 (19,338)	0 (0)	66 (0)	4949 (427)	4949 (18,911)
	PLAX_cc1	4641 (16,968)	0 (0)	3087 (0)	777 (12,093)	777 (4875)
	PLAX_cw1	8306 (18,745)	0 (0)	3634 (0)	2336 (15,258)	2336 (3487)
	PLAX_cw2	9726 (19,741)	0 (0)	0 (0)	4863 (0)	4863 (19,741)
Rocking	PLAX_ra2	10,802 (18,732)	0 (0)	0 (0)	5401 (274)	5401 (18,458)
	PLAX_ra1	8664 (32,771)	0 (868)	3353 (13,900)	4075 (14,416)	1236 (3587)
	PLAX_rt1	8276 (26,316)	0 (29)	932 (2035)	3672 (19,017)	3672 (5235)
	PLAX_rt2	5671 (17,542)	0 (0)	520 (275)	274 (461)	4877 (16,806)
Sliding	PLAX_sm2	10,862 (16,280)	0 (0)	274 (274)	5294 (0)	5294 (16,006)
	PLAX_sm1	4216 (17,108)	0 (274)	4062 (502)	77 (10,593)	77 (5739)
	PLAX_sl1	4675 (21,745)	0 (177)	4151 (768)	524 (14,294)	0 (6506)
	PLAX_sl2	4941 (14,089)	0 (0)	0 (0)	0 (0)	4941 (14,089)
	PLAX_hp	5438 (18,136)	0 (192)	3516 (9120)	1374 (7329)	548 (1495)
	PLAX_lp	6238 (32,060)	0 (0)	3642 (16,248)	1292 (11,654)	1304 (4158)
**(II) Parasternal Short-Axis View**						
			**Quality**			
**Position**		**Total**	**Best**	**Acceptable**	**Poor**	**Bad**
Optimal view	PSAX	53,921 (188,329)	46,560 (148,215)	4662 (23,375)	2699 (16,034)	0 (705)
Rotating	PSAX_cc2	5206 (18,924)	0 (0)	0 (0)	0 (1026)	5206 (17,898)
	PSAX_cc1	6092 (17,149)	0 (0)	625 (743)	4800 (11,309)	667 (5097)
	PSAX_cw1	7076 (20,043)	0 (0)	0 (548)	0 (15,995)	7076 (3500)
	PSAX_cw2	1246 (19,475)	0 (0)	345 (0)	901 (0)	0 (19,475)
Rocking	PSAX_ra2	12,481 (17,979)	0 (0)	201 (0)	6916 (0)	5364 (17,979)
	PSAX_ra1	6669 (20,187)	539 (548)	1346 (4194)	4236 (13,010)	548 (2435)
	PSAX_rt1	4692 (19,124)	345 (512)	4347 (1297)	0 (14,086)	0 (3229)
	PSAX_rt2	5279 (15,894)	0 (0)	0 (0)	5279 (0)	0 (15,894)
Tilting	PSAX_tp2	17,826 (28,072)	0 (0)	0 (548)	8913 (3135)	8913 (24,389)
	PSAX_tp1	8819 (34,166)	5994 (23,855)	1245 (3386)	1031 (5641)	549 (1284)
	PSAX_ta0	3999 (11,690)	0 (0)	425 (337)	3574 (10,805)	0 (548)
	PSAX_ta1	5228 (19,588)	0 (0)	394 (0)	4834 (4594)	0 (14,994)
	PSAX_ta2	4602 (13,963)	0 (0)	0 (0)	4602 (0)	0 (13,963)
Sliding	PSAX_hp2	4911 (17,973)	0 (0)	0 (0)	0 (0)	4911 (17,973)
	PSAX_hp1	11,240 (17,561)	0 (0)	476 (0)	5382 (3684)	5382 (13,877)
	PSAX_hp0	5467 (15,490)	0 (0)	5467 (1466)	0 (11,088)	0 (2936)
	PSAX_lp1	11,091 (33,854)	8665 (18,710)	2426 (3353)	0 (9322)	0 (2469)
	PSAX_lp2	17,596 (29,512)	0 (0)	0 (0)	8798 (2517)	8798 (26,995)
**(III) Apical Four-Chamber View**						
			**Quality**			
**Position**		**Total**	**Best**	**Acceptable**	**Poor**	**Bad**
Optimal view	A4C	38,733 (123,392)	17,228 (53,867)	10,811 (30,277)	9863 (32,544)	831 (6704)
Rotating	A4C_cc2	5530 (18,571)	0 (0)	0 (0)	5530 (0)	0 (18,571)
	A4C_cc1	4727 (17,418)	0 (0)	3421 (0)	1306 (11,052)	0 (6366)
	A4C_cw1	5530 (19,860)	0 (0)	0 (0)	0 (13,079)	5530 (6781)
	A4C_cw2	5418 (13,103)	0 (0)	0 (0)	0 (0)	5418 (13,103)
Tilting	A4C_tp0	3183 (9007)	0 (0)	2414 (0)	769 (6478)	0 (2529)
	A4C_tp1	4304 (14,638)	0 (0)	1895 (0)	2409 (7864)	0 (6774)
	A4C_tp2	4284 (16,666)	0 (0)	0 (0)	4284 (0)	0 (16,666)
Rocking, followed by Rotating /Sliding	A4C_ra_cc	4965 (17,774)	0 (0)	0 (0)	0 (274)	4965 (17,500)
	A4C_ra	5220 (15,561)	0 (0)	548 (1859)	3153 (12,334)	1519 (1368)
	A4C_ra_cw	4444 (12,964)	0 (0)	0 (0)	0 (0)	4444 (12,964)
	A4C_rt_cc	3695 (12,818)	0 (0)	0 (0)	0 (1113)	3695 (11,705)
	A4C_rt	11,543 (41,674)	343 (822)	2390 (13,687)	6036 (15,262)	2774 (11,903)
	A4C_rt_cw	5250 (15,860)	0 (0)	0 (0)	0 (845)	5250 (15,015)
	A4C_rt_sm	7855 (27,049)	0 (0)	0 (0)	377 (2393)	7478 (24,656)
Placing at the midpoint	A4Chw_rt	4753 (19,342)	0 (0)	274 (0)	4479 (2784)	0 (16,558)
	A4Chw	4097 (13,774)	0 (0)	2314 (0)	1783 (8046)	0 (5728)
	A4Chw_ra	4482 (17,289)	0 (0)	0 (0)	0 (778)	4482 (16,511)

Numbers in parentheses represent the combined number of samples used for training and validation.

**Table 5:** Performance Metrics of Position Evaluation Models for Standard Views.

View, Optimal View	Accuracy	Recall	Precision	F1-Score
Parasternal Long-Axis View, PLAX	0.761	0.619	0.751	0.679
Parasternal Short-Axis View, PSAX	0.908	0.926	0.810	0.864
Apical Four-chamber View, A4C	0.900	0.812	0.852	0.831

**Table 6:** Performance Metrics of Quality Evaluation Models for Standard Views.

View	Accuracy	Recall	Precision	F1-Score
Parasternal Long-Axis View	0.740	0.597	0.773	0.674
Parasternal Short-Axis View	0.877	0.813	0.879	0.845
Apical Four-chamber View	0.875	0.746	0.746	0.746

**Table 7:** Comparison of Position-Quality Model Combination and Standalone Position Evaluation Model.

Optimal View	Model	Recall	Precision	F1-Score
PLAX	position * ∩ quality evaluation	0.471	0.850	0.606
	position * ∪ quality evaluation	0.757	0.676	0.714
	Standalone position evaluation	0.619	0.751	0.679
PSAX	position ∩ quality evaluation	0.815	0.857	0.835
	position ∪ quality evaluation	0.964	0.687	0.802
	Standalone position evaluation	0.926	0.810	0.864
A4C	position ∩ quality evaluation	0.602	0.946	0.736
	position ∪ quality evaluation	0.869	0.773	0.818
	Standalone position evaluation	0.812	0.852	0.831

* A ∩ B: Intersection of A and B, A ∪ B: Union of A and B.

**Table 8:** Comparison of Models for Identifying Images Meeting Both Position and Quality Criteria.

Optimal View	Model	Recall	Precision	F1-Score
PLAX	PLAX * ∩ Appropriate Quality	0.493	0.777	0.603
	PLAX * ∪ Appropriate Quality	0.788	0.613	0.690
PSAX	PSAX ∩ Appropriate Quality	0.843	0.843	0.843
	PSAX ∪ Appropriate Quality	0.971	0.657	0.784
A4C	A4C ∩ Appropriate Quality	0.718	0.816	0.764
	A4C ∪ Appropriate Quality	0.937	0.603	0.734

* A ∩ B: intersection of A and B, A ∪ B: union of A and B.

## Data Availability

The datasets generated or analyzed during the current study are held at Hamamatsu University School of Medicine. Access to the data is not publicly available due to institutional and ethical restrictions, but may be granted to qualified researchers upon reasonable request. Data access requests should be submitted to the Hamamatsu University School of Medicine, Next Generation Creative Education Center for Medicine, Engineering, and Informatics (hama-nxcec511@hama-med.ac.jp) and will require prior approval from the committee.

## Supplementary Materials

The following supporting information can be downloaded at: https://www.mdpi.com/article/10.3390/diagnostics16071032/s1, Table S1: Position evaluation criteria; Table S2: Quality evaluation criteria; Table S3: Inference time, model size, and overfitting-related parameters for each backbone architecture; Table S4: Distribution of positions and quality in the main training dataset; Figure S1: Classification results of the position evaluation model for all sections, including non-optimal sections.
