# Supplementary material for: Development of an Artificial Intelligence-Based System for Evaluating Transthoracic Echocardiographic Imaging in Focus Cardiac Ultrasonography
Source: Diagnostics (Basel). 2026 Mar 30;16(7):1032. doi: 10.3390/diagnostics16071032 (PMC13073534; doi:10.3390/diagnostics16071032)

Supplementary Information

Supplementary Table S1 Position evaluation criteria

(I) Parasternal long-axis view

Position	Criteria
**PLAX**	Left atrium (LA), left ventricle (LV), left ventricular outflow tract (LVOT), right ventricle (RV), aortic valve (AV), mitral valve (MV), and interventricular septum (IVS) are visible.
**PLAX_cc2**	The left ventricular cavity becomes even smaller (almost absent).
**PLAX_cc1**	The left ventricular cavity appears smaller, with a false apex and visible chordae tendineae.
**PLAX_cw1**	The left ventricular cavity appears smaller, with a false apex and visible chordae tendineae, accompanied by a small "wine cork" appearance.
**PLAX_cw2**	A cross-section between the long-axis and short-axis views.
**PLAX_ra2**	The anterior mitral leaflet tip is located in the rightmost quarter of the field, and the aortic valve is not visible.
**PLAX_ra1**	The anterior mitral leaflet tip is fully visible on the right half of the field; if the aortic valve is cut off, it is considered poor.
**PLAX_rt1**	The mitral valve is fully visible in the left half of the field.
**PLAX_rt2**	The left end (tip) of the anterior leaflet touches or is cut off at the edge of the field.
**PLAX_sm2**	The left ventricular cavity becomes even smaller (almost absent).
**PLAX_sm1**	The left ventricular cavity becomes smaller, with a false apex and visible chordae tendineae.
**PLAX_sl1**	A small "wine cork" appearance. *
**PLAX_sl2**	A large "wine cork" appearance. *
**PLAX_hp**	The beam is not perpendicular to the LV, with the apex appearing farther from the probe and the aortic root closer. (LV is not horizontal.)
**PLAX_lp**	The beam is not perpendicular to the LV, with the apex appearing closer to the probe and the aortic root farther. (LV is not horizontal.)

*"wine cork" appearance refers to a state where the left ventricular outflow tract (LVOT) region exhibits trapezoidal high echogenicity, resembling the appearance of being sealed with a wine cork.

(II) Parasternal short-axis view

Position	Criteria
**PSAX**	LV, RV, IVS, Anterior leaflet mitral valve (AMVL), Posterior leaflet mitral valve (PMVL) are visible. The LV is as circular as possible. The left ventricular cavity is aligned with the centerline of the field and remains within the inner boundary of the field.
**PSAX_cc2**	The left ventricular wall appears interrupted and elongated horizontally, with the mitral valve resembling a long-axis view.
**PSAX_cc1**	The left ventricular wall appears continuous and elongated horizontally, with the mitral valve resembling a long-axis view.
**PSAX_cw1**	The left ventricular wall appears continuous and elongated vertically, with the mitral valve resembling a four-chamber view.
**PSAX_cw2**	The left ventricular wall appears interrupted and elongated vertically, with the mitral valve resembling a four-chamber view.
**PSAX_ra2**	The left ventricular cavity shifts further to the right, exceeding the inner boundary and extending outside the field.
**PSAX_ra1**	The left ventricular cavity shifts slightly to the right without overlapping the centerline and remains within the inner boundary of the field.
**PSAX_rt1**	The left ventricular cavity shifts slightly to the left without overlapping the centerline and remains within the inner boundary of the field.
**PSAX_rt2**	The left ventricular cavity shifts further to the left, exceeding the inner boundary and extending outside the field.
**PSAX_ta2**	The aortic valve is not visible.
**PSAX_ta1**	The aortic valve is visible.
**PSAX_ta0**	An intermediate view between the aortic and mitral valve levels.
**PSAX_tp1**	The papillary muscles are visible.
**PSAX_tp2**	The papillary muscles are not visible.
**PSAX_hp2**	The aortic valve is not visible.
**PSAX_hp1**	The aortic valve is visible.
**PSAX_hp0**	An intermediate view between the aortic and mitral valve levels.
**PSAX_lp1**	The papillary muscles are visible.
**PSAX_lp2**	The papillary muscles are not visible.

(III) Apical Four-chamber view

Position	Criteria
**A4C**	LA, LV, LVOT, RV, AV, MV, IVS are visible (ideal). It is acceptable if the RV lateral wall is not visible during LV imaging.
**A4C_cc2**	RA and RV cavities disappear.
**A4C_cc1**	RA and RV cavities become smaller.
**A4C_cw1**	LA and LV cavities become smaller.
**A4C_cw2**	LA and LV cavities disappear.
**A4C_tp0**	The five chambers (RA, RV, LA, LV, Aorta (Ao)) are visible.
**A4C_tp1**	LA disappears.
**A4C_tp2**	Both RA and LA disappear.
**A4C_ra_cc**	The LV lateral wall is near the right edge of the field, with RA and RV cavities disappearing.
**A4C_ra**	The LV lateral wall is near the right edge of the field.
**A4C_ra_cw**	The LV lateral wall is near the right edge of the field, with LA and LV cavities disappearing.
**A4C_rt_cc**	The RV lateral wall is near the left edge of the field, with RA and RV cavities disappearing.
**A4C_rt**	The RV lateral wall is near the edge of the field.
**A4C_rt_cw**	The RV lateral wall is near the left edge of the field, with LA and LV cavities disappearing.
**A4C_rt_sm**	The apex is cut off at the right edge of the field.
**A4Chw_ra**	The lengths of the IVS and IAS are equal (1:1), with the RA and RV lateral walls near the right edge of the field.
**A4Chw**	The lengths of the IVS and interatrial septum (IAS) are equal (1:1).
**A4Chw_rt**	The lengths of the IVS and IAS are equal (1:1), with the RA and RV lateral walls near the left edge of the field.

Supplementary Table S2 Quality evaluation criteria

(I) Parasternal long-axis view

Quality	Criteria
**Best**	LA, LV, LVOT, RV, AV, MV, and IVS are visible, and chordae tendineae are not continuously depicted.
**Acceptable**	The beam is not perpendicular to the LV (LV appears tilted or not horizontal). A false apex is visible. One structure is invisible during either systole or diastole. Chordae tendineae appear intermittently.
**Poor**	Any one of LA, LV, LVOT, RV, AV, MV, or IVS is not visible.
**Bad**	Two or more of LA, LV, LVOT, RV, AV, MV, or IVS are not visible.

(II) Parasternal short-axis view

Quality	Criteria
**Best**	LV, RV, IVS, and MV are visible, with the LV appearing circular and centrally aligned within the field.
**Acceptable**	LV is not perfectly circular. In diastole, the LV is circular, but in systole, it resembles a snowman shape (intermediate view between mitral and aortic valve levels).
**Poor**	Any one of LV, RV, IVS, AMVL, or PMVL is not visible. RV appears reduced by more than half its normal size. In diastole, the LV resembles a snowman shape, and the aortic valve is not visible.
**Bad**	Portions of the anterior, lateral, or inferior LV walls are cut off. Two or more of LV, RV, IVS, AMVL, or PMVL are not visible. Two or more of LA, LV, RA, RV, IVS, MV, TV, or IAS are not visible. IVS and IAS lengths are equal (1:1), with a septal tilt of approximately 20° or more.

(III) Apical Four-chamber view

Quality	Criteria
**Best**	LA, LV, RA, RV, IVS, MV, TV, and IAS are visible (ideal). Even if the RV lateral wall is not visible, all other structures are depicted. In diastole, the LV lateral wall is visible (RV wall may be partially missing).
**Acceptable**	In systole, the LV lateral wall is visible (RV wall may be partially missing). The apex is slightly truncated but the LV lateral wall remains visible (artifact). Septal tilt is approximately 20° or more.
**Poor**	Any one of LA, LV, RA, RV, IVS, MV, TV, or IAS is not visible. IVS and IAS lengths are equal (1:1). The LV lateral wall is not visible in systole. The aortic valve (Ao) is visible, indicating a five-chamber view.
**Bad**	The LV apex is not centrally located within the field (frame-out). Two or more of LA, LV, RA, RV, IVS, MV, TV, or IAS are not visible. IVS and IAS lengths are equal (1:1), with a septal tilt of approximately 20° or more.

Supplementary Table S3 Inference time, model size, and overfitting-related parameters for each backbone architecture

Backbone architecture	Inference time, mean (SD) [ms]	Parameters (M)	Model size [MB]	Validation F1 score
efficientnet_b1	15.24 (1.39)	6.52	25.82	0.99737
efficientnet_b2	17.25 (2.38)	7.71	30.55	0.99737
nfnet_f0	24.70 (6.50)	68.43	273.70	0.41839
mobilenetv2_050	9.03 (1.17)	0.69	2.69	0.99784
mobilenetv2_100	8.99 (1.07)	2.23	8.77	0.99689
mobilenetv2_110d	11.87 (1.34)	3.24	12.77	0.99761
mobilevit_xxs	15.73 (1.67)	0.95	3.79	0.99665
mobilevit_xs	17.23 (1.59)	1.93	7.70	0.99486
mobilevit_s	18.49 (1.84)	4.94	19.71	0.99784
mobilevitv2_050	23.04 (3.43)	1.11	4.43	0.99737
mobilevitv2_075	25.98 (1.78)	2.48	9.88	0.99952
mobilevitv2_100	29.97 (2.09)	4.39	17.50	0.99641

Experiments were conducted on a MacBook Pro with Apple M1 Pro GPU using PyTorch with Metal Performance Shaders (MPS) backend.
Dataset size was 1,335 KB for all experiments.

Supplementary Table S4 Distribution of positions and quality in the main training dataset

(I) Parasternal Long Axis view			Quality			
		total	**best**	**acceptable**	**poor**	**bad**
Position	**PLAX**	14,089	7,985	3,911	2,027	166
	**PLAX_cc2**	14,089	0	0	302	13,787
	**PLAX_cc1**	14,089	0	0	10,075	4,014
	**PLAX_cw1**	14,089	0	0	11,492	2,597
	**PLAX_cw2**	14,089	0	0	0	14,089
	**PLAX_ra2**	14,089	0	0	204	13,885
	**PLAX_ra1**	14,089	358	6,002	6,214	1,515
	**PLAX_rt1**	14,089	16	1,098	10,220	2,755
	**PLAX_rt2**	14,089	0	208	375	13,506
	**PLAX_sm2**	14,089	0	238	0	13,851
	**PLAX_sm1**	14,089	217	423	8,716	4,733
	**PLAX_sl1**	14,089	117	496	9,281	4,195
	**PLAX_sl2**	14,089	0	0	0	14,089
	**PLAX_hp**	14,089	140	7,054	5,744	1,151
	**PLAX_lp**	14,089	0	7,152	5,083	1,854

(Ⅱ) Parasternal Short Axis view			Quality			
		total	**best**	**acceptable**	**poor**	**bad**
Position	**PSAX**	11,690	9,210	1,452	970	58
	**PSAX_cc2**	11,690	0	0	645	11,045
	**PSAX_cc1**	11,690	0	519	7,709	3,462
	**PSAX_cw1**	11,690	0	306	9,347	2,037
	**PSAX_cw2**	11,690	0	0	0	11,690
	**PSAX_ra2**	11,690	0	0	0	11,690
	**PSAX_ra1**	11,690	318	2,388	7,579	1,405
	**PSAX_rt1**	11,690	319	787	8,606	1,978
	**PSAX_rt2**	11,690	0	0	0	11,690
	**PSAX_tp2**	11,690	0	221	1,306	10,163
	**PSAX_tp1**	11,690	8,176	1,176	1,915	423
	**PSAX_ta0**	11,690	0	337	10,805	548
	**PSAX_ta1**	11,690	0	0	2,712	8,978
	**PSAX_ta2**	11,690	0	0	0	11,690
	**PSAX_hp2**	11,690	0	0	0	11,690
	**PSAX_hp1**	11,690	0	0	2,433	9,257
	**PSAX_hp0**	11,690	0	1,114	8,356	2,220
	**PSAX_lp1**	11,690	6,475	1,181	3,170	864
	**PSAX_lp2**	11,690	0	0	990	10,700

(Ⅲ) Apical Four-chamber			Quality			
		total	**best**	**acceptable**	**Poor**	**bad**
Position	**A4C**	9,007	3,964	2,135	2,428	480
	**A4C_cc2**	9,007	0	0	0	9,007
	**A4C_cc1**	9,007	0	0	5,715	3,292
	**A4C_cw1**	9,007	0	0	6,003	3,004
	**A4C_cw2**	9,007	0	0	0	9,007
	**A4C_tp0**	9,007	0	0	6,478	2,529
	**A4C_tp1**	9,007	0	0	4,817	4,190
	**A4C_tp2**	9,007	0	0	0	9,007
	**A4C_ra_cc**	9,007	0	0	151	8,856
	**A4C_ra**	9,007	0	1,066	7,140	801
	**A4C_ra_cw**	9,007	0	0	0	9,007
	**A4C_rt_cc**	9,007	0	0	782	8,225
	**A4C_rt**	9,007	173	2,965	3,329	2,540
	**A4C_rt_cw**	9,007	0	0	486	8,521
	**A4C_rt_sm**	9,007	0	0	793	8,214
	**A4Chw_ra**	9,007	0	0	403	8,604
	**A4Chw**	9,007	0	0	5,234	3,773
	**A4Chw_rt**	9,007	0	0	1,260	7,747

Supplementary Figure S1 Classification results of the position evaluation model for all sections, including non-optimal sections

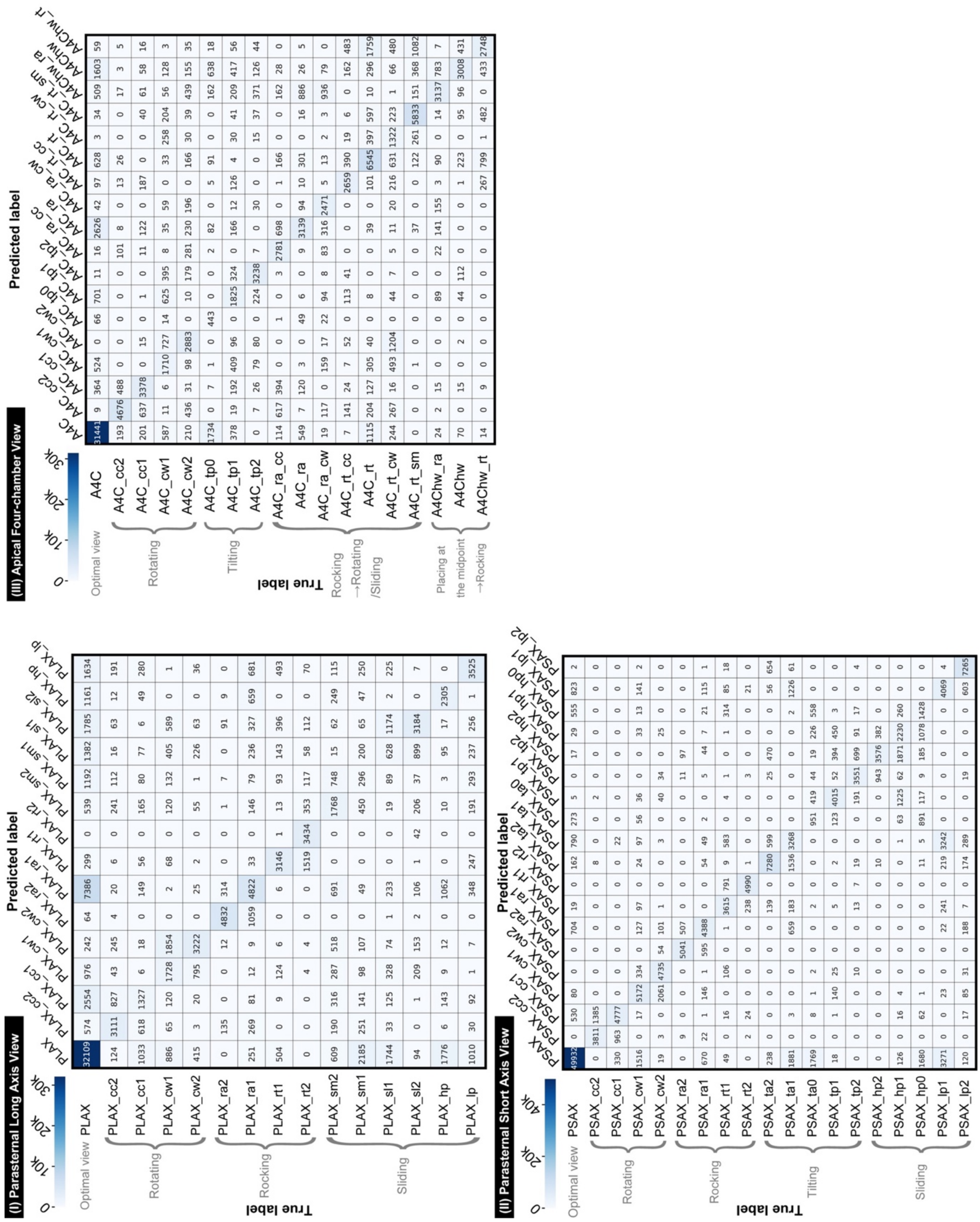

Supplement: Supplementary file 1 [file diagnostics-16-01032-s001.zip › diagnostics-4130127-supplementary.pdf]
